# Systematic review and network meta-analysis of outcomes of transcatheter strategies and surgical shunts for treatment of duct-dependent cyanotic congenital heart disease

**DOI:** 10.3389/fcvm.2025.1594779

**Published:** 2025-09-02

**Authors:** Qiao Zhi Chee, Santosi Buvaneswarran, Ching Kit Chen, Liang Shen, Swee Chye Quek

**Affiliations:** ^1^ Division of Cardiology, Department of Paediatrics, Khoo Teck Puat-National University Children’s Medical Institute, National University Health System, Singapore, Singapore; ^2^ Department of Neonatology, Khoo Teck Puat-National University Children’s Medical Institute, National University Health System, Singapore, Singapore; ^3^ Department of Paediatrics, Yong Loo Lin School of Medicine, National University of Singapore, Singapore, Singapore; ^4^ Biostatistics Unit, Yong Loo Lin School of Medicine, National University of Singapore, Singapore, Singapore

**Keywords:** congenital, paediatric, cyanotic, shunt, stent, transcatheter

## Abstract

**Introduction:**

Cyanotic congenital heart disease with duct-dependent pulmonary blood flow requires initial palliation before definitive repair. Traditionally, these were surgical systemic-to-pulmonary shunts (SPS) such as the modified Blalock-Taussig-Thomas shunt. Transcatheter procedures are recently emerging as alternative options. Previous reviews have compared ductal stenting (DS) or right ventricular outflow tract (RVOT) intervention to surgical SPS, but none compared all three strategies in a single meta-analysis.

**Methods:**

We performed a systematic review and network meta-analysis for a three-way comparison of DS, RVOT intervention, and surgical SPS as initial palliation in children aged 0–3 with cyanotic congenital heart disease. Primary outcomes were mortality rates, complication rates, and re-intervention rates. MEDLINE, EMBASE, and Cochrane databases were searched for publications from 2003 to 2023. References of previous systematic reviews were screened.

**Results:**

Twenty publications were included for analysis, for a total of 4,441 patients. Transcatheter options consistently ranked superior to surgical SPS for Early and Overall Mortality. DS also outperformed surgical SPS for Procedural Complications. Surgical SPS was worst for Early Re-interventions, but outperforms both transcatheter options for Overall Re-interventions and Intra-procedural Mortality. Both DS and RVOT stenting conferred a shorter overall hospitalization duration compared to surgical SPS, and DS also gave a shorter length of stay in intensive care compared to surgical SPS.

**Conclusions:**

Transcatheter options are viable, safe, and attractive alternatives to traditional surgical shunts in the treatment of duct-dependent cyanotic heart disease. With wider adoption, increased collective expertise will help to further improve patient outcomes of these less-invasive techniques.

**Systematic Review Registration:**

PROSPERO (CRD42024489748).

## 1 Introduction

Cyanotic congenital heart disease with duct-dependent pulmonary blood flow often requires an initial palliation to allow somatic growth before definitive repair. Traditionally, a surgical systemic-to-pulmonary shunt (SPS) is created to secure a more reliable source of pulmonary blood flow, with the modified Blalock-Taussig-Thomas shunt (mBTS) being the most common.

Transcatheter procedures are emerging as options to a surgical approach (1). One such strategy is ductal stenting (DS), where a stent is placed in the native ductus arteriosus to keep it patent without having a continuous intravenous prostaglandin infusion. This secures a medium-term solution while avoiding the need for immediate surgery.

More recently, right ventricular outflow tract (RVOT) intervention by stenting or ballooning has been explored as another strategy, where the native right ventricular outflow tract is augmented to improve antegrade pulmonary blood flow, thus eliminating the need for a surgical shunt.

Previous reviews have compared either DS or RVOT intervention against the traditional surgical SPS, but there is none comparing all three strategies against each other in a single systematic review or meta-analysis (2–7). The aim of this study is to conduct a systematic review and meta-analysis for a three-way comparison of the safety and efficacy of two transcatheter options (DS and RVOT intervention) and surgical SPS.

## 2 Methods

This systematic review and meta-analysis was conducted and reported in accordance with the standards established in the Cochrane Handbook for Systematic Reviews of Interventions and Preferred Reporting Items for Systematic Review and Meta-Analysis (PRISMA) statement (8). The methodology of this paper was published *pre-hoc* in the International Prospective Register of Systematic Reviews (PROSPERO) registry (CRD42024489748).

### 2.1 Eligibility criteria

Publications were included for review if they met the following inclusion criteria: English language, full-length articles, human subjects aged 0 to 3 years, comparative study of at least one transcatheter option (DS or RVOT intervention) against SPS as a control group, and described at least one outcome of interest.

Publications which were not a comparative study were excluded (such as case reports, case series, topical reviews, and opinion pieces), as were abstracts, oral presentations, and conference proceedings without a full-length article. While systematic reviews and meta-analyses were excluded from the analysis itself, their references were manually searched for possible source of relevant publications.

### 2.2 Sources of information

A comprehensive literature search of MEDLINE and EMBASE databases was conducted. All publications from January 2003 to December 2023 inclusive were included. Additionally, the Cochrane Database of Systematic Reviews was searched for relevant systematic reviews.

### 2.3 Search strategy

The search strategy was developed by two reviewers (CQZ and SB) in consensus. The search syntax for MEDLINE and EMBASE were constructed to replicate the search logic as similarly as possible. The complete search syntax may be found in Table 1.

**Table 1:** Search syntax for MEDLINE and EMBASE databases.

No.	Query
	MEDLINE:
8	#1 AND ((#2 AND #4) OR [#3 AND (#4 OR #5)]) AND #6 AND #7 AND 2003:2023[dp]
7	([“Heart Defects, Congenital”(Mesh)] OR “Tetralogy of Fallot”[Mesh]) OR “Ventricular Outflow Obstruction”[Mesh] OR “cyanotic congenital” OR “cyanotic heart” OR “cyanotic CHD” OR “CCHD”
6	“Blalock-Taussig Procedure”[Mesh] OR Blalock* OR “BT shunt” OR “mBTS” OR “Pulmonary Artery/surgery”[Mesh] OR “systemic pulmonary shunt”[tiab:∼2]
5	“Angioplasty, Balloon”[Mesh] OR balloon*
4	“Stents”[Mesh] OR stent*
3	“right ventricular outflow” OR RVOT
2	[“Ductus Arteriosus”[Mesh] OR “Ductus Arteriosus, Patent”[Mesh] OR PDA OR ductus OR ductal OR “ductus arteriosus” OR “arterial duct”]
1	[“Child, Preschool”[Mesh] OR “Infant”[Mesh] OR “Infant, Newborn”[Mesh] OR “children” OR “pediatric” OR “paediatric” OR “infant” OR “newborn”] AND “Humans”[Mesh]
Final expanded query: (“child, preschool”[MeSH Terms] OR “Infant”[MeSH Terms] OR “infant, newborn”[MeSH Terms] OR “children”[All Fields] OR “pediatric”[All Fields] OR “paediatric”[All Fields] OR “Infant”[All Fields] OR “newborn”[All Fields]) AND “Humans”[MeSH Terms] AND (((“Ductus Arteriosus”[MeSH Terms] OR “ductus arteriosus, patent”[MeSH Terms] OR “PDA”[All Fields] OR “ductus”[All Fields] OR “ductal”[All Fields] OR “Ductus Arteriosus”[All Fields] OR “arterial duct”[All Fields]) AND [“Stents”[MeSH Terms] OR “stent*”[All Fields]]) OR ([“right ventricular outflow”[All Fields] OR “RVOT”[All Fields]] AND (“Stents”[MeSH Terms] OR “stent*”[All Fields] OR [“angioplasty, balloon”[MeSH Terms] OR “balloon*”[All Fields]]))) AND (“Blalock-Taussig Procedure”[MeSH Terms] OR “blalock*”[All Fields] OR “BT shunt”[All Fields] OR “mBTS”[All Fields] OR “pulmonary artery/surgery”[MeSH Terms] OR “systemic pulmonary shunt”[Title/Abstract:∼2]) AND (“heart defects, congenital”[MeSH Terms] OR “Tetralogy of Fallot”[MeSH Terms] OR “Ventricular Outflow Obstruction”[MeSH Terms] OR “cyanotic congenital”[All Fields] OR “cyanotic heart”[All Fields] OR “cyanotic CHD”[All Fields] OR “CCHD”[All Fields]) AND 2003/01/01:2023/12/31[Date—Publication]	
	EMBASE:
#12	#1 AND #6 AND #7 AND #11 AND [2003-2023]/py
#11	#8 OR #10
#10	#3 AND #9
#9	#4 OR #5
#8	#2 AND #4
#7	“cyanotic heart disease”/exp OR “fallot tetralogy”/exp OR “heart outflow tract obstruction”/syn OR “cyanotic congenital” OR “cyanotic heart” OR “cyanotic chd” OR cchd
#6	blalock taussig shunt”/syn OR blalock* OR “bt shunt” OR mbts OR (“systemic” NEAR/2 “pulmonary” NEAR/2 “shunt”)
#5	angioplasty'/syn OR “balloon”/syn OR balloon*
#4	stent'/syn OR stent*
#3	heart right ventricle outflow tract'/syn OR “right ventricular outflow” OR rvot
#2	ductus arteriosus’/syn OR “patent ductus arteriosus”/syn OR pda OR ductus OR ductal OR “arterial duct”
#1	(“infant”/syn OR “child”/syn OR children OR pediatric OR paediatric OR infant OR newborn) AND “human”/syn
Final expanded query: [(“infant”/syn OR “child”/syn OR children OR pediatric OR paediatric OR infant OR newborn) AND “human”/syn] AND [“blalock taussig shunt”/syn OR blalock* OR “bt shunt” OR mbts OR (“systemic” NEAR/2 “pulmonary” NEAR/2 “shunt”)] AND (“cyanotic heart disease”/exp OR “fallot tetralogy”/exp OR “heart outflow tract obstruction”/syn OR “cyanotic congenital” OR “cyanotic heart” OR “cyanotic chd” OR cchd) AND ([(“ductus arteriosus”/syn OR “patent ductus arteriosus”/syn OR pda OR ductus OR ductal OR “arterial duct”) AND (“stent”/syn OR stent*)] OR ((“heart right ventricle outflow tract”/syn OR “right ventricular outflow” OR rvot) AND [(“stent”/syn OR stent*) OR (“angioplasty”/syn OR “balloon”/syn OR balloon*)])) AND [2003-2023]/py	

The following search terms were used as free text, or where available, equivalent standard MeSH and Emtree terms: human; paediatric or children; ductus arteriosus and stent; right ventricular outflow tract, and stent or balloon; cyanotic congenital heart disease or Tetralogy of Fallot; Blalock-Taussig shunt or systemic-to-pulmonary shunt.

### 2.4 Data extraction

Two reviewers (CQZ and SB) screened titles and abstracts of the search results for publications relevant to study question. Where the eligibility of the publication was unclear from the title or abstract, the full text was retrieved for screening. Duplicate publications were removed, and data from included publications were extracted for further analysis.

The following outcomes were sought from each publication: Overall and Early Mortality, Overall and Early Re-intervention rate, Procedural Complications, Intra-procedural Mortality, time to second-stage surgery, length of overall hospitalization, and length of stay in intensive care unit (ICU).

As the exact criteria for defining the “Early” period varied across publications, for the purpose of our review, mortality and re-intervention outcome was considered “Early” if it occurred: within 30 days of the initial intervention, within the same admission, or considered by study authors to be directly related to the initial procedure. “Overall” referred to any instance of the outcome reported by the publication, regardless of duration of follow-up. Procedural Complications referred to complications relating to the initial procedure, as reported by each publication. Time to second-stage surgery or definitive surgery was recorded as the same outcome.

### 2.5 Data synthesis

Where mean and standard deviation of study groups were not directly reported, these statistics were estimated from the statistics reported by each publication, using the Data Estimation and Conversion for Meta-Analysis (DECoMA) tool (Cochrane Taiwan) (9, 10). Meta-analysis and network meta-analysis were conducted with Stata 18.0 software package (StataCorp). Two-tailed alpha was set at 0.05. Adjusted point estimates were pooled using the generic inverse variance method under the fixed-effect model.

Network meta-analysis allowed simultaneous comparison of all three interventions by direct and indirect evidence. The mean rank and surface under the cumulative ranking (SUCRA) of each intervention was calculated for each outcome of interest. Local incoherence was tested by node-splitting, while global incoherence was tested using Design-by-Treatment model to check for the entire network. The overall heterogeneity was assessed using the Chi-square test, with a threshold of *p* < 0.1 for significant heterogeneity. Papers that might cause significant heterogeneity were identified and removed from the network meta-analysis.

### 2.6 Quality appraisal

Quality appraisal of each included publication was performed by two reviewers (CQZ and SB) with the relevant risk-of-bias tool (Newcastle-Ottawa Scale was used for retrospective or prospective cohorts) (11).

### 2.7 Dispute resolution

Non-consensus between the two reviewers was resolved by a third senior author (CCK).

## 3 Results

### 3.1 Publication selection

Total of 337 publications were identified from initial search of each database, and 30 duplicate publications removed. From the remaining, 270 publications were excluded during the initial screening of titles and abstracts for relevance. Of 37 left, the following exclusions were made: 2 (non-English language), 6 (not full-length articles), 1 (non-comparative study), 8 (study groups and outcomes were not relevant). Furthermore, 2 studies were identified as a subgroup analysis of a larger study already being included, and hence were removed from analysis (12, 13).

**Figure 1:**
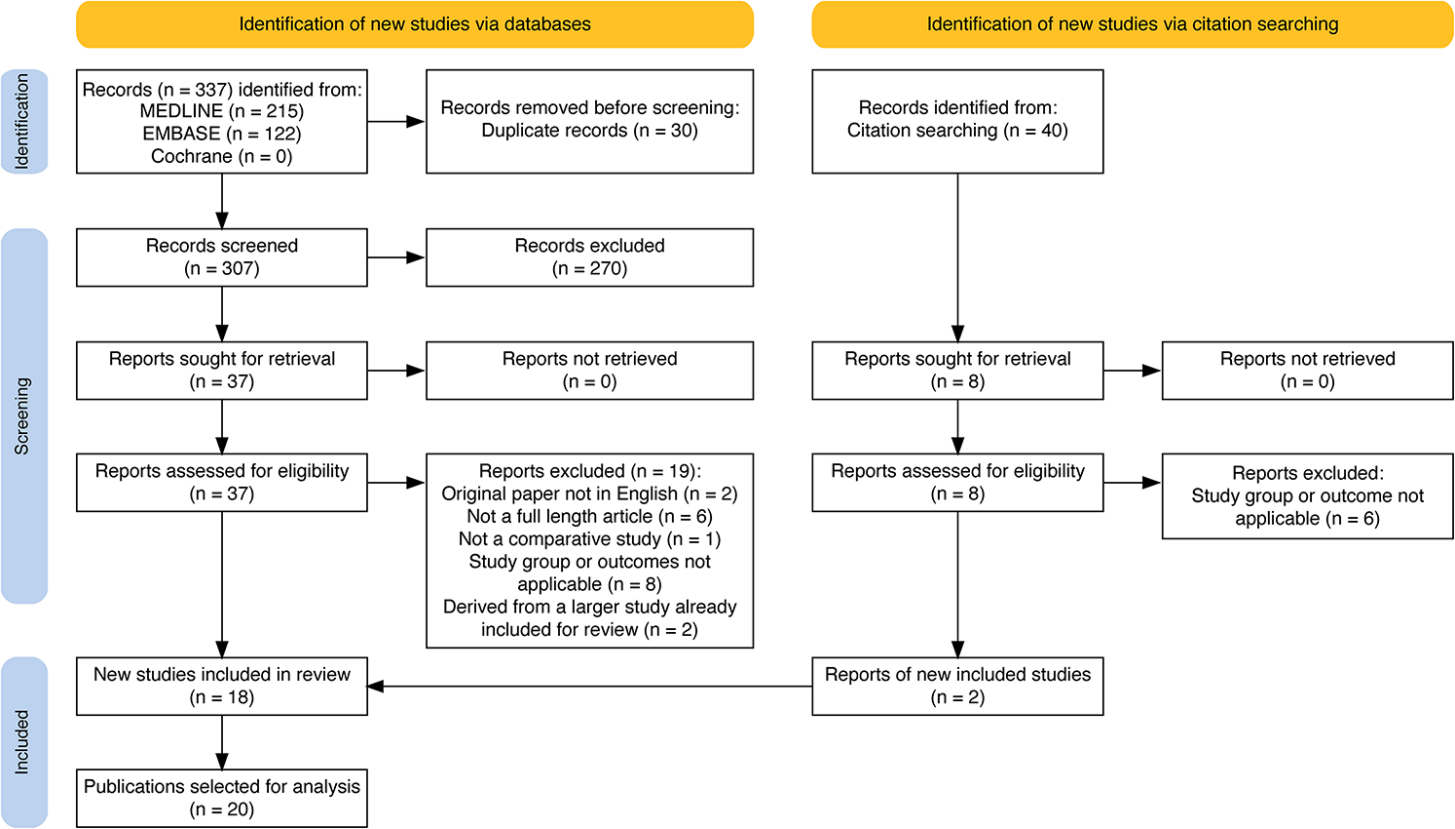
PRISMA flow diagram for selection of publications for analysis.

Additionally, 40 publications were identified for screening from the references of relevant systematic reviews (2–7),—from which 2 publications were eventually identified as eligible for inclusion. In effect, a total of 20 publications were selected for analysis (14–33). The PRISMA flow diagram is reported in Figure 1.

### 3.2 Study characteristics

The basic characteristics of each included publication are detailed in Table 2 (14–33). Among the 20 selected publications, 15 publications compared DS against SPS and 4 studies compared RVOT intervention against SPS. A single study compared all three modalities of treatment.

**Table 2:** Study characteristics of publications selected for analysis.

Authors/year of publication	Study site	Study design	Sample Size, *n*	Cardiac lesions	Patients in each group			Outcomes Reported
					SPS *(n),* (type/s of SPS)	DS (*n)*	RVOT (*n)*	
McMullan D.M, et al, 2014 (25)	USA	Retrospective review	55	DORV, TOF, PA, Ebstein, TA, complex TGA, unbalanced AVSD	42, (mBTS)	13		30-day mortality Overall mortality (12 m or until 2nd stage) Procedural complications Procedural death 30-day re-intervention Overall re-intervention Time to second stage/repair
Quandt D., et al, 2017 (28)	UK	Retrospective review	101	TOF	41 (mBTS)	60		Procedural mortality Overall mortality (12 m or until 2nd stage) Procedural complications Procedural death (intraprocedural/24 h) Overall re-intervention LOS (ICU) LOS (entire stay)
Ho A.B., et al, 2022 (21)	UK	Retrospective review	87	TOF, DORV, Ebstein, PA, TA, TGA/VSD/PS, AVSD, univentricular anatomy	29 (mBTS)	36	22	30-day mortality Overall mortality (until repair/end of follow up period) Procedural complications or “serious adverse events” intra-procedural mortality Re-intervention in first 7 days Overall re-intervention Time to second stage/repair
Alhawri K.A., et al, 2019 (15)	Ireland	Retrospective review	10	Complete AVSD	2 (mBTS)		8	Overall mortality (until end of follow up) Overall re-intervention
Santoro G., et al, 2009 (31)	Italy	Retrospective review	136	TOF, PA, complex CHD with PA/PS	87 (mBTS)	49		In-hospital and procedural mortality Procedural/surgical morbidity
Prabhu N.K., et al, 2021 (27)	USA	Retrospective review	34	PA-IVS, unbalanced AVSD, TA, others	23 (mBTS)	11		30-day operative mortality Overall mortality to stage 2 Procedural death 90-day re-intervention Overall re-intervention Time to second stage/repair LOS (ICU) LOS (entire stay)
Lekchuensakul S., et al, 2022 (22)	Thailand	Retrospective review	98	Single or biventricular anatomy with PA/PS	64 (mBTS)	34		Overall in-hospital mortality Overall Mortality (until definitive repair) Procedural Complications Procedural Death Early Re-intervention (within-admission) Overall Re-intervention LOS (entire stay)
Rocchia F., et al, 2022 (30)	Argentina	Retrospective review	20	TOF	11 (mBTS)		9	Overall mortality Procedural complications LOS (entire stay)
Lemley B.A., et al, 2022 (23)	USA	Retrospective review	1,874	PS and others, excluding HLHS	1,340 (mBTS)	534		30-day mortality Overall mortality Overall re-intervention LOS (ICU) LOS (entire stay)
Sheth S., et al, 2023 (32)	USA	Retrospective review	52	Ductal dependent pulmonary blood flow	38 (mBTS)	14		Inpatient mortality Overall re-intervention LOS (entire stay)
Valencia E., et al, 2023 (33)	USA	Retrospective review	936	CHD with ductal-dependent pulmonary blood flow (individual lesions not specified)	610 (mBTS)	326		Inpatient mortality 30-day re-intervention 6-month re-intervention LOS (entire stay)
Aurigemma D., et aql, 2018 (17)	USA	Retrospective review	27	PA-VSD	22 (mBTS or central shunt)		5	Inpatient mortality Overall mortality (until repair) 24-hour re-intervention Overall re-intervention LOS (entire stay)
Glatz A.C., et al, 2018 (19)	USA	Retrospective review	357	PA-IVS,PA-VSD, VSD/PS, isolated PS	251 (mBTS)	106		Mortality (until 3 years) Procedural complications Overall re-intervention Time to second stage/repair LOS (ICU) LOS (entire stay)
Bentham J.R., et al, 2018 (18)	UK	Prospective cohort	254	CHD with ductal-dependent pulmonary blood flow (individual lesions not specified)	171 (mBTS)	83		30-day mortality Overall mortality (until stage 2) Overall re-intervention Time to second stage/repair Los (picu) Los (entire stay)
Ratnayaka K., et al, 2021 (29)	USA	Retrospective review	88	TOF, PA-IVS, PA, PS, TGA/PS, Heterotaxy syndrome, PA/VSD, Ebstein anomaly, isolated PA	41 (mBTS)	47		Operative mortality (30-day or during hospitalization) Overall mortality (operative and inter-stage) Procedural complications Overall re-intervention
Helal A.M., et al, 2022 (20)	Saudi Arabia	Retrospective review	134	PA-IVS, VSD/PS, PA-VSD, TA, isolated PS	51 (mBTS)	83		(No data specific for short term) Overall (hospital and inter-stage) mortality Procedural complications Time to second stage/repair LOS (PICU) LOS (entire stay)
Al Kindi H., et al, 2023 (14)	Oman	Retrospective review	71	PA-VSD, TOF, TGA/PA, TGA with PA-VSD or PS, PA-IVS, TA, AVSD	38 (mBTS)	33		Overall mortality (until 3 years) Overall re-intervention
Mallula K., et al, 2015 (24)	USA	Retrospective review	29	PA-IVS	16 (mBTS)	13		Procedure-related mortality Overall mortality (follow up period) Procedural complications Intra-procedural mortality Early re-intervention (same admission) Overall re-intervention LOS (entire stay)
Amoozgar H, et al, 2012 (16)	Iran	Retrospective review	35	TOF, PA, TA, AVSD, PA-VSD, PA-IVS, TGA with PS	20 (mBTS or central shunt)	15		Procedural mortality Overall mortality (6 m)
Nasser BA, et al, 2019 (26)	Saudi Arabia	Prospective cohort	43	Patients who underwent procedures for augmentation of pulmonary blood flow (individual lesions not specified)	10 (mBTS)	33		28-day mortality Overall mortality (until 6 months) Intra-procedural mortality Overall re-intervention LIS (ICU)

SPS, surgical systemic-to-pulmonary shunt; DS, ductal stenting; RVOT, right ventricular outflow tract intervention; AVSD, atrioventricular septal defect; CHD, congenital heart disease; DORV, double-outlet right ventricle; ICU, intensive care unit; IVS, intact ventricular septum; mBTS, modified Blalock-Taussig-Thomas shunt; PA, pulmonary atresia; PS, pulmonary stenosis; TA, tricuspid atresia; TGA, transposition of great arteries; TOF, tetralogy of fallot; VSD, ventricular septal defect; UK, United Kingdom; USA, United States of America.

A total of 4,441 patients were analysed across all publications. Patients underwent intervention from 0 to 11 months of age. Surgical SPS included not only the most common modified Blalock-Taussig-Thomas shunt, but also central aorto-pulmonary shunts. All RVOT interventions were performed via RVOT stenting. Among patients with a reported cardiac diagnosis, 352 of 3,694 patients were classified as Tetralogy of Fallot. The baseline characteristics of the included patients are presented in Table 3.

**Table 3:** Study characteristics of publications selected for analysis.

Population characteristics	SPS Group (*n* = 2,907)	DS Group (*n* = 1,430)	RVOT Group (*n* = 104)
Mean age, *days* (*SD*)	25 (240)	12 (8)	36 (18)
Mean weight, *kg* (*SD*)	3.4 (0.4)	3.2 (0.2)	3.1 (0.6)
Male sex, *n* (*%*)	1,258 (43.2%)	531 (37.1%)	39 (37.5%)
Preterm, *n* (*%*)	273 (9.4%)	195 (13.6%)	21 (20.2%)

SPS, surgical systemic-to-pulmonary shunt; DS, ductal stenting; RVOT, right ventricular outflow tract intervention; SD, standard deviation.

Out of the 20 publications, all reported data for Overall Mortality, while 17 reported data for Early Mortality. Overall Re-intervention data was reported in 16 publications, while 6 reported data for Early Re-intervention. Procedural Complication rates was recorded from 10 publications, and Intra-procedural Mortality from 11 publications.

Network meta-analysis was performed for indirect comparisons and ranking of treatment modalities. There was sufficient data for network meta-analysis for the outcomes of Overall Mortality, Early Mortality, Intra-procedural Mortality, Procedural Complications, Early Re-interventions, and Overall Re-interventions. All network meta-analyses were closed loops, with at least one direct comparison between each treatment modality.

The results of risk of bias assessment done using Newcastle-Ottawa Scale (11), are presented in Table 4.

**Table 4:** Risk of bias assessment of publications selected for analysis by Newcastle-Ottawa scale (10).

Publication	Domain	Overall risk of bias	Outcome	
	Selection	Comparability		
McMullan, 2014 (25)	★★★★	–	★★★	Low
Quandt, 2017 (28)	★★★★	–	★★★	Low
Ho, 2022 (21)	★★★★	–	★★★	Low
Alhawri, 2019 (15)	★★★★	–	★★★	Low
Santoro, 2009 (31)	★★★★	–	★★★	Low
Prabhu, 2021 (27)	★★★★	–	★★★	Low
Lekchuensakul, 2022 (22)	★★★	★	★★★	Low
Rocchia, 2022 (30)	★★★★	–	★★★	Low
Lemley, 2022 (23)	★★★★	★★	★★★	Low
Sheth, 2023 (32)	★★★★	★	★★★	Low
Valencia, 2023 (33)	★★★★	–	★★★	Low
Aurigemma, 2018 (17)	★★★★	–	★★★	Low
Glatz, 2018 (19)	★★★★	★★	★★★	Low
Bentham, 2018 (18)	★★★★	★★	★★★	Low
Ratnayaka, 2021 (29)	★★★★	–	★★★	Low
Helal, 2022 (20)	★★★★	–	★★★	Low
Al Kindi, 2023 (14)	★★★★	–	★★★	Low
Mallula, 2015 (24)	★★★★	–	★★★	Low
Amoozgar, 2012 (16)	★★★★	–	★★★	Low
Nasser, 2019 (26)	★★★	–	★★★	Unclear

### 3.3 Primary outcomes

**Figure 2:**
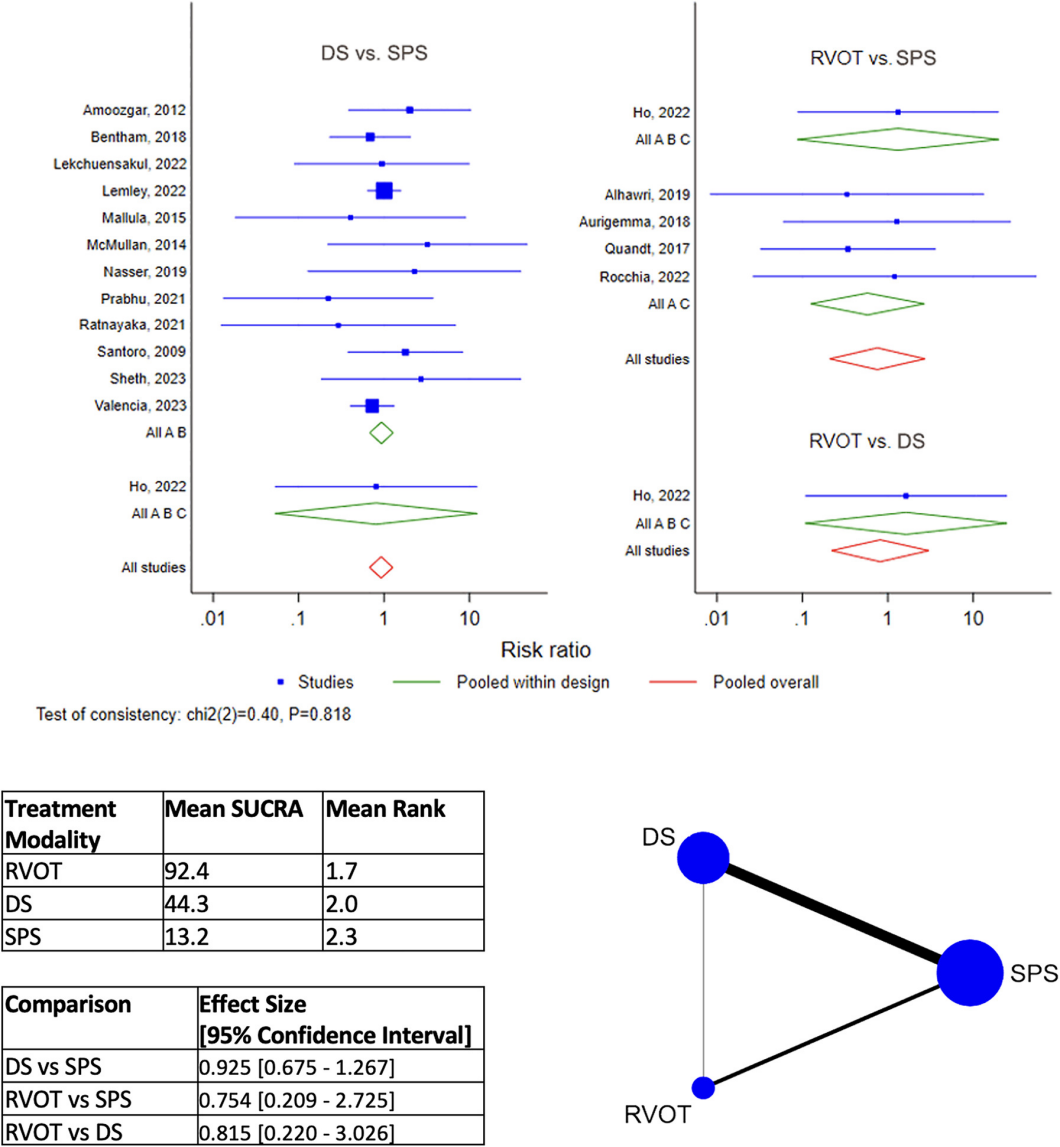
Results of network meta-analysis for early mortality. (SPS: Surgical systemic-to-pulmonary shunt; DS: ductal stenting; RVOT: right ventricular outflow tract intervention; SUCRA: surface under the cumulative ranking).

**Figure 3:**
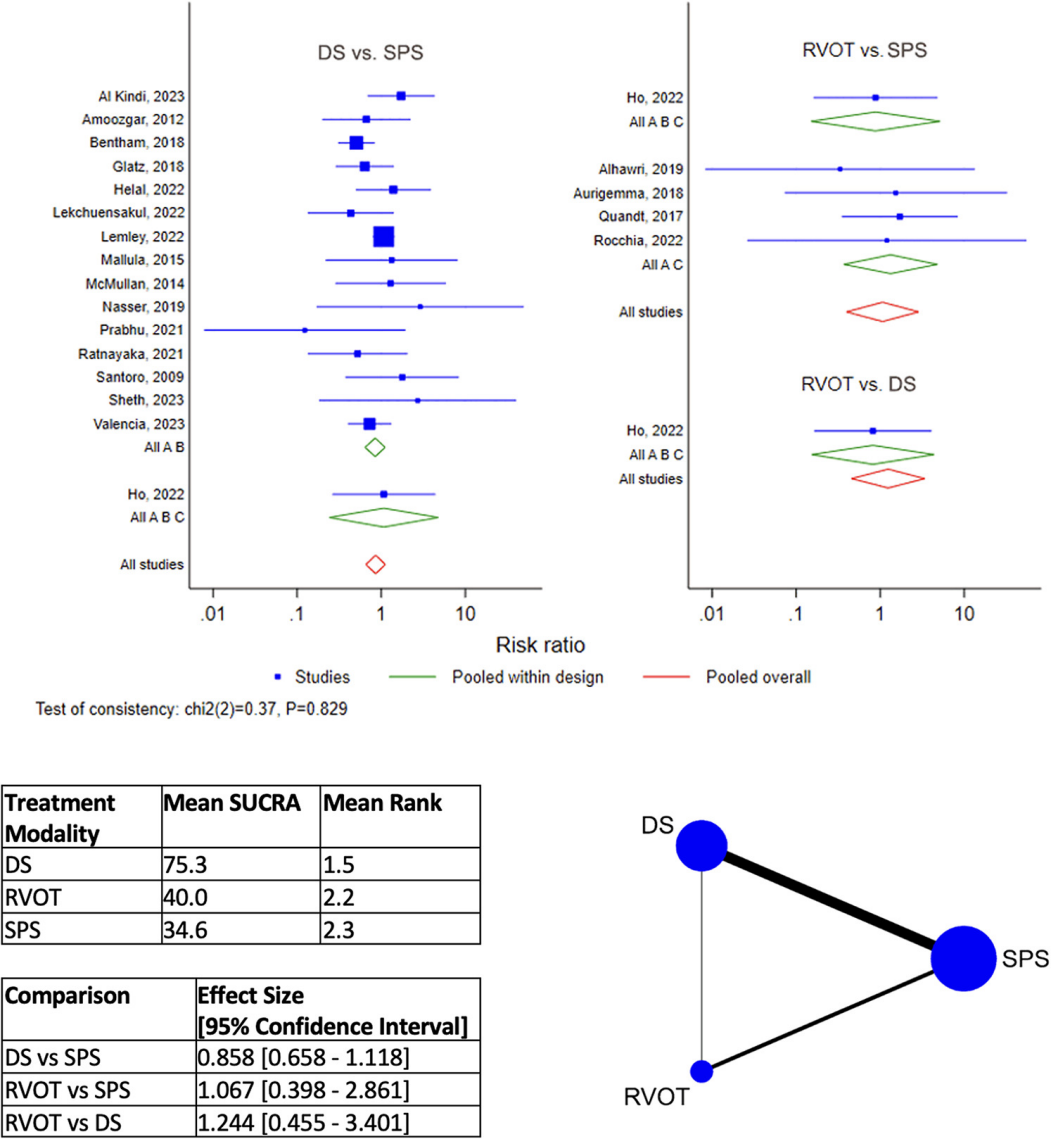
Results of network meta-analysis for overall mortality. (SPS: Surgical systemic-to-pulmonary shunt; DS: ductal stenting; RVOT: right ventricular outflow tract intervention; SUCRA: surface under the cumulative ranking).

By network meta-analysis, RVOT intervention was ranked as best modality for the outcome of Early Mortality (Figure 2), and DS was best for Overall Mortality (Figure 3). Surgical SPS was ranked the poorest modality for both Early and Overall Mortality. However, surgical SPS was the best-ranked modality for Intra-procedural Mortality (mean rank 1.5; *cf.* DS 2.2 and RVOT 2.3). There was no statistically significant differences between pairwise comparisons of each treatment modality for both Early Mortality and Overall Mortality (Figuress 2,3).

**Figure 4:**
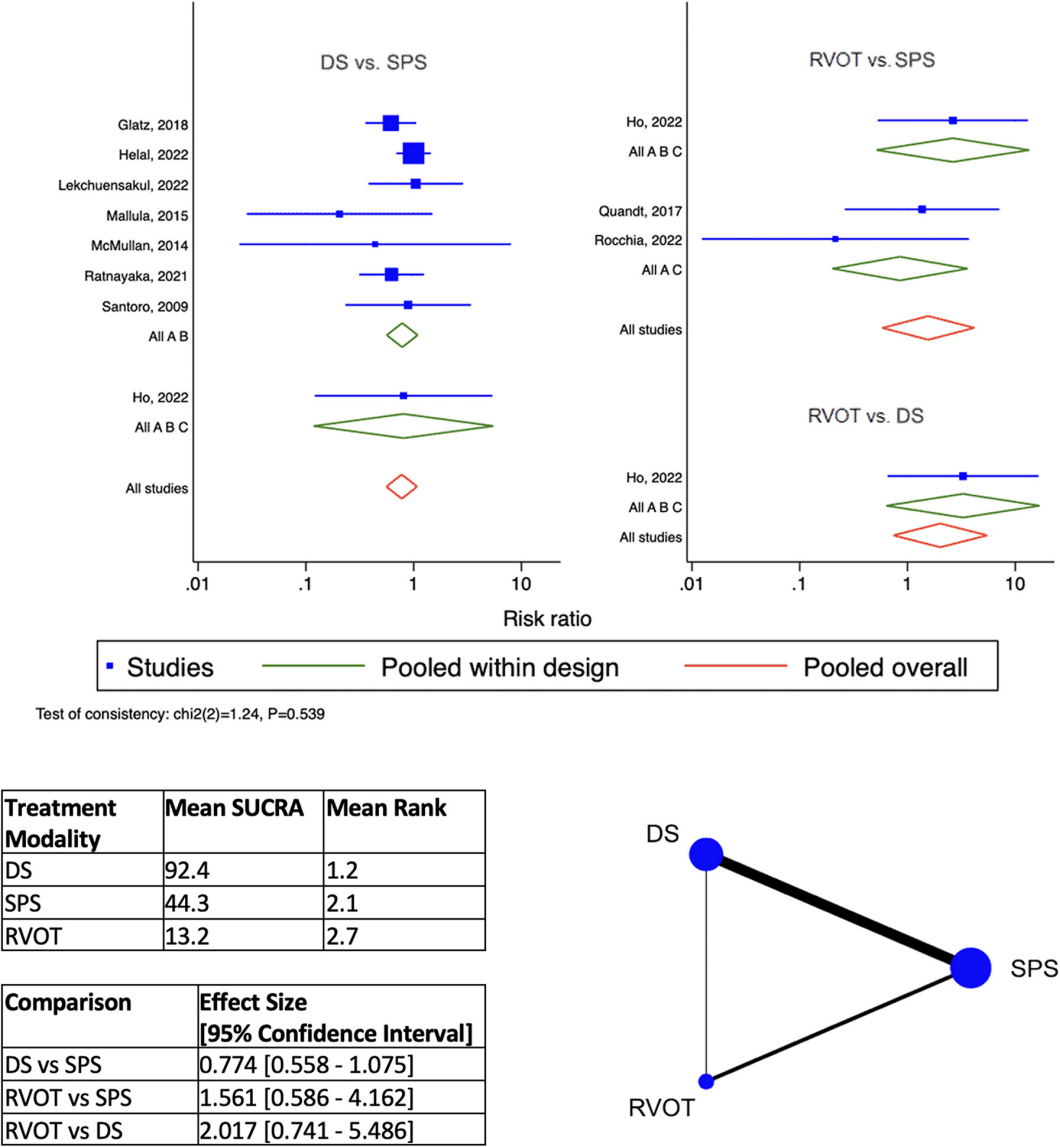
Results of network meta-analysis for procedural complications. (SPS: Surgical systemic-to-pulmonary shunt; DS: ductal stenting; RVOT: right ventricular outflow tract intervention; SUCRA: surface under the cumulative ranking).

**Figure 5:**
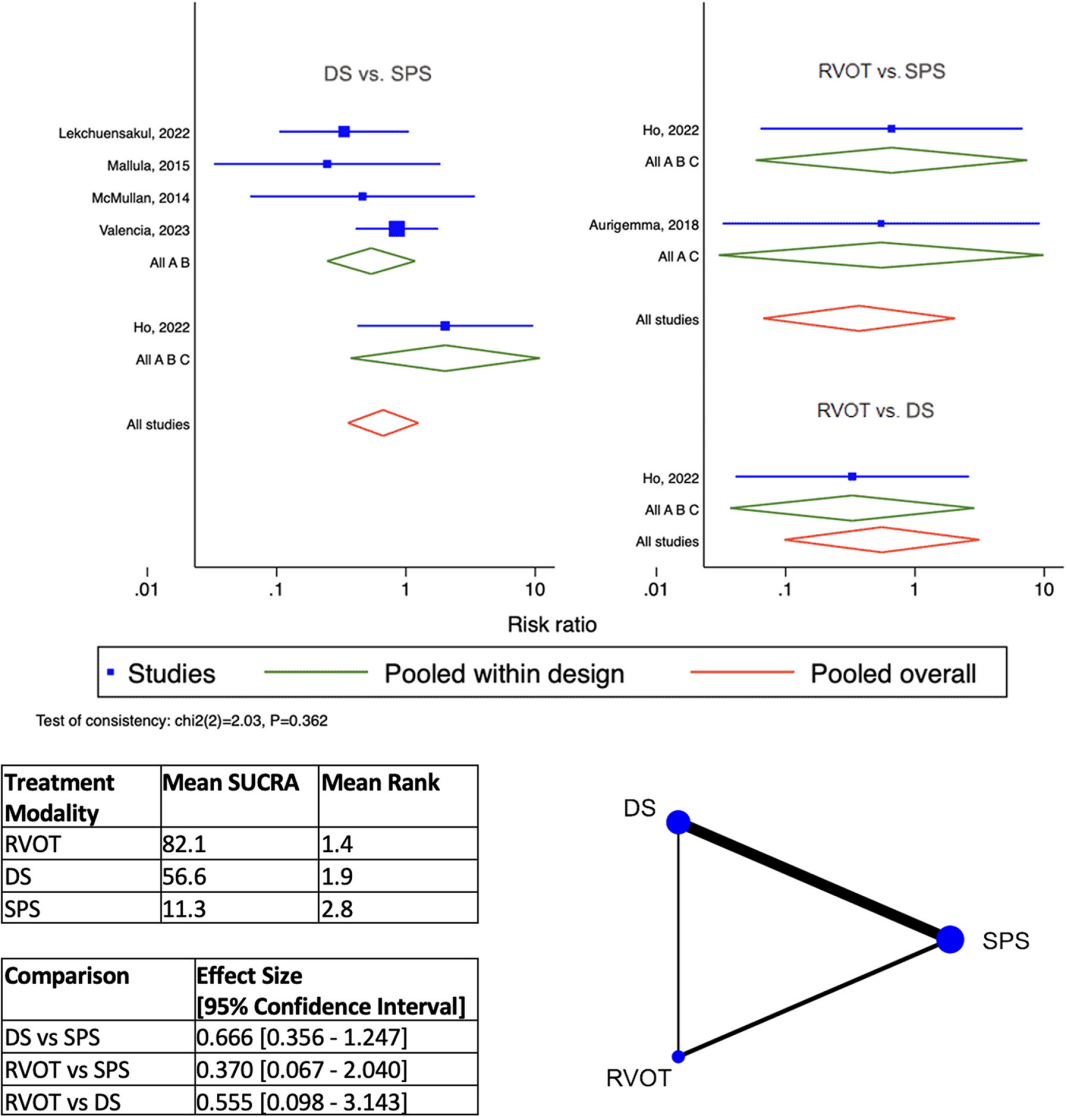
Results of network meta-analysis for early Re-intervention. (SPS: Surgical systemic-to-pulmonary shunt; DS: ductal stenting; RVOT: right ventricular outflow tract intervention; SUCRA: surface under the cumulative ranking).

**Figure 6:**
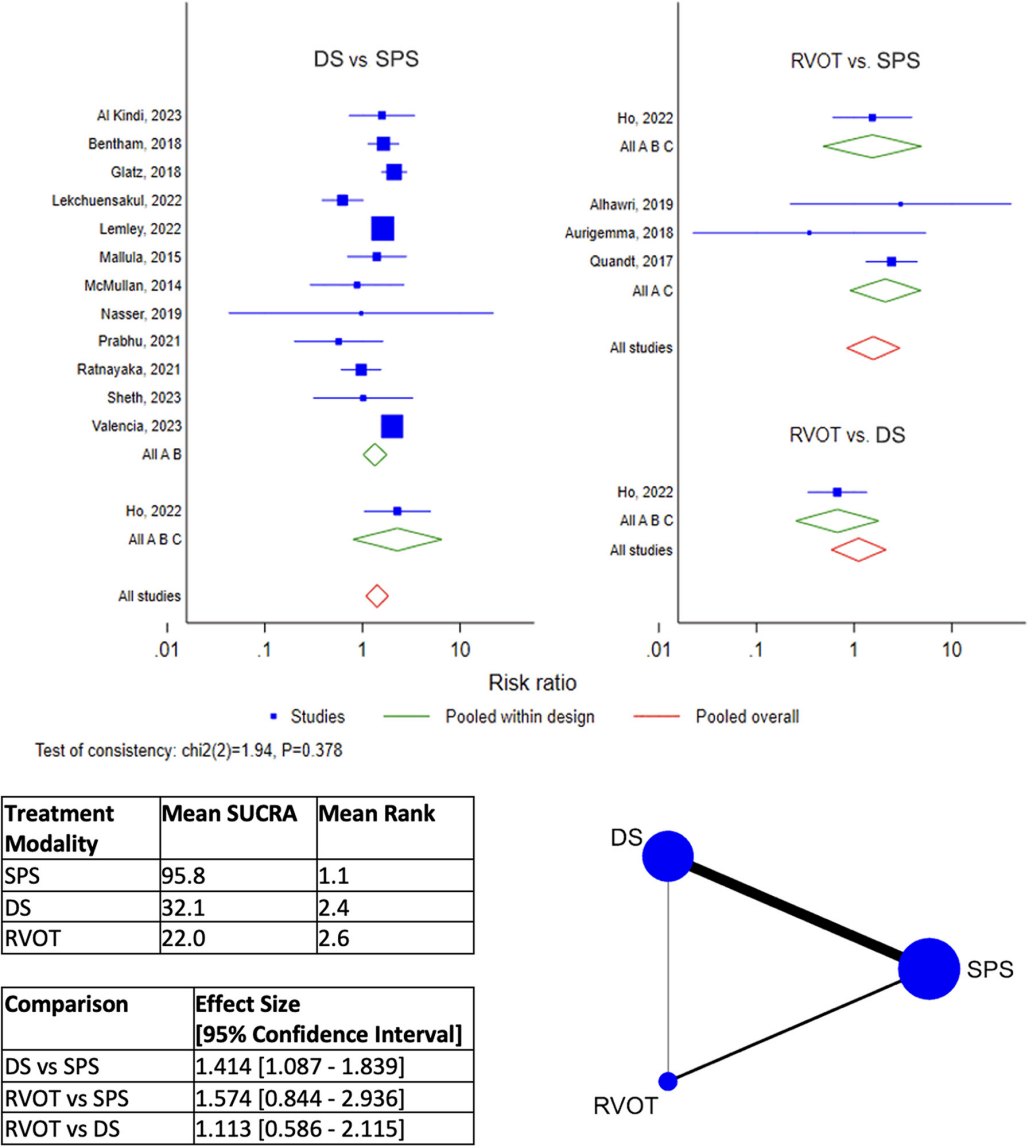
Results of network meta-analysis for overall Re-intervention. (SPS: Surgical systemic-to-pulmonary shunt; DS: ductal stenting; RVOT: right ventricular outflow tract intervention; SUCRA: surface under the cumulative ranking).

DS is the best-ranked modality for Procedural Complications (Mean SUCRA = 92.5, Mean Rank = 1.2), by network meta-analysis (Figure 4). RVOT intervention was worst-ranked for Procedural Complications (Mean SUCRA = 13.2, Men Rank = 2.7). Surgical SPS was worst-ranked by network meta-analysis for Early Re-intervention rate (Mean SUCRA = 11.3, Mean Rank = 2.8) (Figure 5), yet was best-ranked for Overall Re-intervention rate (Mean SUCRA = 95.8, Mean Rank = 1.1) (Figure 6).

### 3.4 Secondary outcomes

There was no difference in time to second-stage surgery (or definitive repair) between DS or surgical SPS.

**Figure 7:**
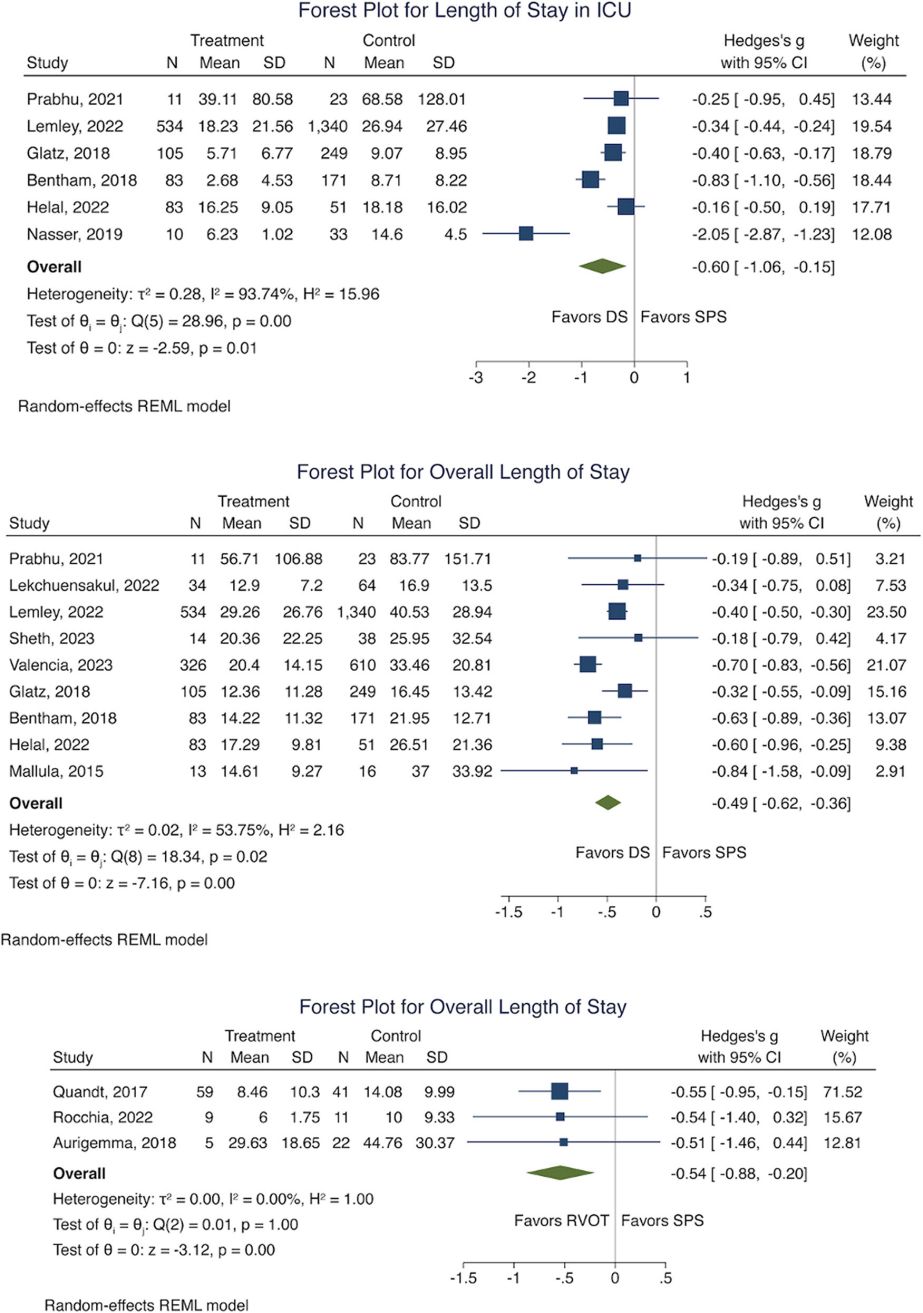
Forest plot for, length of stay in ICU (DS Vs SPS) and oveall length of stay (DS Vs SPS, RVOT Vs SPS). (SPS: Surgical systemic-to-pulmonary shunt; DS: ductal stenting; RVOT: right ventricular outflow tract intervention; ICU: intensive care unit).

Patients who underwent DS compared to surgical SPS as initial treatment had a shorter ICU stay [mean difference −0.60 (−1.06, −0.15)] (Figure 7). Both DS and RVOT intervention conferred a shorter overall hospitalization duration compared to surgical SPS, with results slightly favouring RVOT intervention over DS [RVOT vs. SPS mean difference −0.54 [−0.88 to −0.20]; DS vs. SPS mean difference −0.49 [−0.62 to −0.36]] (Figure 7).

There was insufficient data to perform meta-analysis of RVOT intervention against SPS for time to second-stage surgery and length of ICU stay. Due to small sample sizes, network meta-analysis of secondary outcomes was not performed.

**Figure 8:**
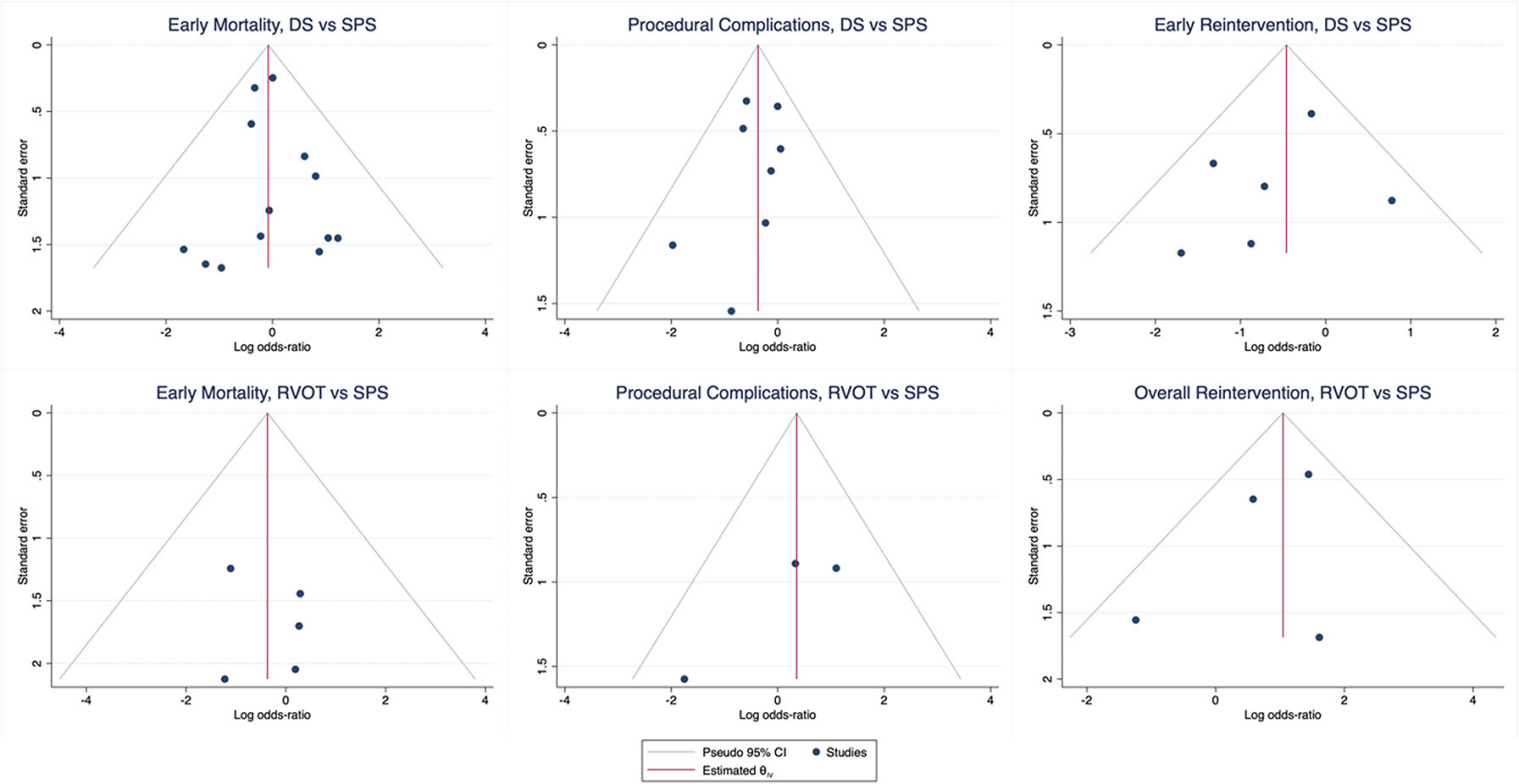
Funnel plots of early outcome, procedural complications, and early Re-intervention for pairwise comparison of DS vs. SPS and RVOT vs. SPS. (SPS: Surgical systemic-to-pulmonary shunt; DS: ductal stenting; RVOT: right ventricular outflow tract intervention).

Funnel plots for pairwise outcomes of Early Mortality, Procedural Complications, and Early Re-intervention did not demonstrate significant asymmetry (Figure 8). These were chosen as they were deemed the most directly consequent adverse outcomes of each intervention. Funnel plots of RVOT vs. SPS was not shown as there was only a single publication with this respective direct comparison. Egger regression-based testing confirmed no statistically significant asymmetry, suggesting the absence of publication bias.

## 4 Discussion

Since the introduction of the Blalock-Taussig-Thomas shunt as a pioneering treatment for cyanotic heart disease, there had been significant progress with the development of less-invasive transcatheter options. The growing body of literature on ductal stenting reflects increasing interest in these techniques, particularly for cases where the placement of a surgical shunt is considered risky or even unfeasible.

More recently, the emergence of RVOT stenting has garnered attention, with publications beginning to describe local experiences and outcomes. However, the paucity of large studies highlights the overall limited experience with this newer technique in a relatively rare condition. Where the obstruction is primarily in the RVOT, stenting would in theory address the issue at its root cause, restoring a more physiological pulmonary blood supply compared to ductal stenting, especially in those with difficult or unfavourable ductal anatomy. This provides flexibility in treatment and enables a tailored approach aimed at improving patient outcomes.

As cyanotic heart disease is overall uncommon, and advanced trans-catheter modalities may not be as widely available, unbiased direct comparisons are hard to come by. Our study is the first to compare these three different techniques both directly and indirectly by means of network meta-analysis, addressing the limitation of smaller sample sizes limiting conventional pairwise meta-analysis, (especially for RVOT intervention).

While pairwise comparisons showed no clear advantage of any single modality for Early or Overall Mortality, transcatheter options consistently ranked superior to surgical SPS. RVOT intervention outperformed DS in Early Mortality but not Overall Mortality. The short term outcomes may reflect nature of the procedure, whereas longer term outcomes are likely influenced by the severity and complexity of the underlying heart condition. The discrepancy between early and late outcomes of RVOT intervention may be due to delayed complications, such as re-stenosis of the RVOT over time. Moreover, RVOT stent placement may make subsequent surgical repair technically more challenging. The emergence of bioresorbable stents for RVOT intervention offers a potential solution by not leaving behind foreign material. Although the successful deployment of bioresorbable RVOT stents for paediatric Tetralogy of Fallot has been reported, this remains novel, with concerns about their radial force, resorption rate and cases of early stent failure reported in other use cases (34).

DS has demonstrated a lower rate of Procedural Complications than surgical shunt placement. In patients who underwent DS, the most commonly reported complications, excluding access-related, were stent thrombosis (19 instances in 74 patients) and stent migration (18 instances in 74 patients).

To address the risk of thrombosis, drug-eluting stents present a possible solution, especially when combined with anti-platelet therapy. Although bare-metal stents remain the standard choice for most, a few studies have reported the use of DES. While our study did not perform a direct comparison between bare-metal and drug-eluting stents, accumulating experience with DES may reveal whether they reduce rates of stent thrombosis over time. This is an area of potential interest for future research.

The most common complication in RVOT intervention was RVOT perforation. Although this complication was infrequent, its associated morbidity is high, often requiring emergency surgery or resuscitation. In at least one instance, intra-procedural death was directly attributed to RVOT perforation. Additionally, acute valvular injury has been rarely reported. In one case, the tricuspid valve was injured during stent delivery, and in another, the aortic valve was injured when the stent migrated into the aortic arch.

While the overall complication rate of RVOT intervention remains high, this may reflect the learning curve associated with a new procedure, with rates likely to improve over time. Given the potential for serious complications, it is essential to have a cardiac surgical team on standby during RVOT intervention.

While transcatheter options offer a less invasive strategy, they come with a high rate of re-intervention, primarily due to issues with stent patency. Transcatheter options fared better for Early Re-intervention rates than surgical SPS, suggesting that stent failure tended to occur in a mid-to-long-term time frame. For transcatheter re-interventions, the existing stent or shunt was often balloon dilated or re-stented. In some cases, the native pulmonary arteries were also balloon dilated or stented to improve pulmonary blood flow. The rate of surgical vs. transcatheter re-intervention varies between studies, reflecting the preferences and experience of individual centres.

Despite the promise of transcatheter options, surgical SPS still has a relevant role in the current landscape of treatment strategies. In the setting of a failed transcatheter procedure, a surgical approach would be the necessary fallback.

Our study demonstrated additional advantages of transcatheter modalities, including a shorter ICU stay and reduced overall hospitalization duration compared to surgical approaches. These findings suggest that, as less-invasive options, transcatheter techniques have the potential to match, or even surpass, traditional surgical methods in some outcomes. With further experience and technical refinement, transcatheter options could increasingly offer a viable, effective alternative to surgery for treating cyanotic congenital heart disease.

### 4.1 Limitations

Due to the rarity of cyanotic congenital heart disease, almost all included studies were retrospective cohorts. There were no randomised studies comparing one intervention against another, and therefore the outcomes are subject to selection bias. Among studies, there remained ambiguity in the definition of “complications”, making this outcome subject to each study's interpretation. There was also significant variability in follow-up durations, and lost-to-follow-up rates were generally unreported, introducing further biases that are difficult to address through statistical adjustments alone.

The greatest limitation is the scarcity of comparative data between RVOT intervention compared to other interventions. In published literature, only 4 studies directly compared RVOT intervention with SPS, and only 1 study performed a three-way comparison. We have attempted to mitigate this limitation in our study by use of network meta-analysis and indirect comparisons.

The patient population selected for RVOT intervention was generally older than the patients selected for DS (Table 3), which limits the comparability of outcomes between the two transcatheter intervention groups.

### 4.2 Conclusion

Innovative advances in interventional options for cyanotic congenital heart disease are promising, and continue to offer exciting alternatives to treatment. However, the choice of initial intervention remains highly dependent on the training and experience of each centre, as well as patient-specific factors, such as the native anatomy. Tailoring the intervention to both the institution's expertise and the unique anatomical needs of each patient is key to optimizing outcomes.

This study lends further credibility to transcatheter options as viable, safe, and attractive alternatives to traditional surgical shunts in the treatment of duct-dependent cyanotic heart disease. Our findings suggest that, with continued experience and refinement, especially in techniques like RVOT stenting, transcatheter approaches could see wider adoption and potentially lead to further improvements in patient outcomes. Expanding the use of these less-invasive methods may also help build collective expertise, paving the way for optimized care in this complex patient population.

## Data Availability

The original contributions presented in the study are included in the article/Supplementary Material, further inquiries can be directed to the corresponding author/s.
